# Cat and dog ownership during/after the first year of life and risk for sensitization and reported allergy symptoms at age 13

**DOI:** 10.1002/iid3.267

**Published:** 2019-08-29

**Authors:** Chaifa Al‐Tamprouri, Barman Malin, Hesselmar Bill, Bråbäck Lennart, Sandin Anna

**Affiliations:** ^1^ Department of Clinical Science, Paediatrics Umeå University Umeå Sweden; ^2^ Department of Biology and Biological Engineering, Food and Nutrition Science Chalmers University of Technology Gothenburg Sweden; ^3^ Department of Paediatrics, Institute of Clinical Sciences, Sahlgrenska Academy University of Gothenburg Gothenburg Sweden; ^4^ Section of Sustainable Medicine, Department of Public Health and Clinical Medicine Umeå University Umeå Sweden

**Keywords:** allergic symptoms, asthma, birth cohort, cat‐keeping, dog‐keeping, sensitization

## Abstract

**Background:**

Avoidance of pets as a strategy for preventing atopic diseases has been questioned. This study aimed to identify the risk of sensitization and allergic symptoms at age 13 in relation to dog‐ and cat‐keeping during and after the first year of life.

**Methods:**

The study included all children born at Östersund Hospital in Northern Sweden between February 1996 and January 1997 (n = 1231). At inclusion, parents were asked to answer questionnaires about lifestyle, including cat‐ and dog‐keeping. Dog allergy, cat allergy, hay fever, and asthma were diagnosed based on parental reported allergic symptoms at 13 years of age (n = 834). The risks of sensitization or allergy in relation to dog‐ and cat‐keeping during and after the first year of life were analyzed with logistic regression. To adjust for reverse causation, all subjects that had reported avoidance of pets due to allergic symptoms of the child or allergy in the family (n = 177) were excluded.

**Results:**

Dog‐ or cat‐keeping during the first year of life reduced the risk of sensitization to dog or cat allergens, respectively, and to birch and to at least one of the 10 allergens tested. Cat‐keeping, both during and after the first year of life, reduced the risk of cat allergy and hay fever. Having a dog at home during the first year of life reduced the risk of dog and cat allergy, whereas dog‐keeping after the first year of life did not affect allergic symptoms.

**Conclusions:**

Cat ownership, either during or after the first year of life, may be a strategy for preventing the development of cat allergy and hay fever later in life. Dog ownership reduced the risk of sensitization to dog and birch allergen, and also the risk of cat and dog allergy, but had no effect on hay fever.

## 1 INTRODUCTION

Allergic diseases have increased drastically in Western countries during the past decades. One possible explanation for this is provided by the hygiene hypothesis, which states that a reduced exposure to microorganisms early in life might increase the risk of developing allergic diseases.^1^, ^2^, ^3^ In line with this hypothesis, studies have shown that children living on livestock farms have a lower prevalence of asthma,^4^ rhinoconjunctivitis, and sensitization^5^, ^6^ than other children from the same area. Also, children in regular contact with farms present fewer symptoms of asthma and allergy.^7^ Other lifestyle and environmental factors that may affect the risk of allergy are the number of older siblings, lifestyle, socioeconomic status, and frequency of infections.^8^, ^9^ Having pets at home has also been suggested to reduce the risk of allergy in both children^8^, ^10^, ^11^, ^12^ and adults.^13^ However, the impact of cat and dog ownership on sensitization and the development of allergic symptoms is still debated. Cat‐keeping has been reported both to decrease^8^, ^12^ and increase^14^ the risk of sensitization development. Also, cat‐keeping has been found to be inversely correlated to sensitization to cat allergens and to asthma development.^12^ Dog‐keeping at home during early childhood has been directly associated with less sensitization and inversely associated with asthma later in life.^8^, ^10^, ^14^

Early sensitization to food and inhaled allergens is one of the risk factors for the development of atopic eczema and allergy, a risk factor that can be affected by early preventive strategies.^15^, ^16^ Another risk factor for asthma 17 and allergic symptoms is a parental history of allergy.^18^ Persistence or remission of asthma during childhood is determined by sex, asthma phenotype, and level of sensitization.^19^ It has also been suggested that the immune system needs the right incentives and a strong immune stimulation during infancy to mature and develop tolerance towards harmless allergens.^20^, ^21^

By having access to a prospective birth cohort, we were able to study the relationship between dog‐ and cat‐keeping during and after the first year of life and the development of sensitization and allergic symptoms at 13 years of age. The aim of this study was to quantify and analyze the risk of sensitization, allergy, and allergic respiratory symptoms at age 13 in relation to dog‐ and cat‐keeping *during* the first year of life as well as *after* the first year of life.

## 2 METHODS

### 2.1 Study design

The BAS cohort (BarnAllergiStudien or Paediatric Allergy Study) was designed to prospectively investigate the development of allergy during childhood. The BAS cohort includes all children living in Jämtland in northern Sweden and born at Östersund hospital from February 1996 to January 1997 (n = 1231). Families were enrolled either at their antenatal clinic or at delivery.

Parents answered questionnaires when their child was one (n = 1043, 85%) and 13 (n = 834, 68%) years of age. Questions asked about pet keeping, symptoms of asthma and other allergies, allergy treatments, and whether the household avoided pet keeping due to allergy among other family members.^10^, ^22^ Questions related to allergy symptoms were based on the International Study of Asthma and Allergies in Childhood questionnaire (ISAAC).^23^

### 2.2 Selection of subjects

**Figure 1:**
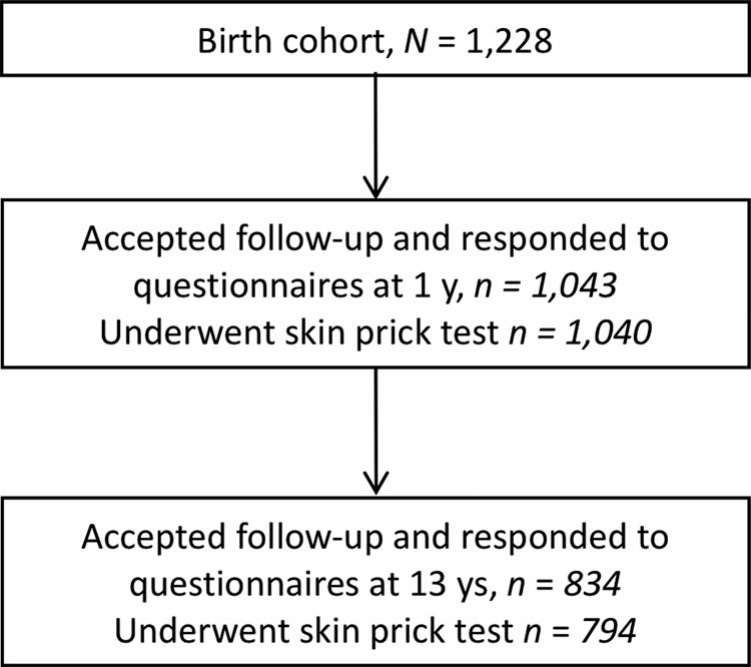
Flow chart of the study population

The population analyzed in this paper include all children who participated in follow‐up at both one and 13 years of age (n = 834) (Figure 1). Altogether, 252 of 833 (30%) of the families had dogs and 179 of 833 (22%) had cats at home during the child's first year of life. Of those that had a dog or a cat at home during the first year of life, 77 subjects had both cat and dog at home. See Table 1 for more characteristics.

**Table 1:** Characteristics of the study population (n = 834^a^)

	Number	Percent
Characteristics^b^		
Sex (boys)	407/809	50
Older sibling	484/782	62
Maternal smoking^c^	93/771	12
Heredity^d^		
Maternal asthma	114/830	14
Maternal hay fever	168/829	20
Paternal asthma	95/819	12
Parental hay fever	163/819	20
Cat‐keeping^d^		
Never cat	460/833	55
Cat‐keeping during first year of life^e^	179/833	22
Cat‐keeping only after first year of life^f^	194/833	23
Dog‐keeping^d^		
Never dog	375/833	45
Dog‐keeping during first year of life^e^	252/833	30
Dog‐keeping only after first year of life^f^	206/833	25

^a^ Included in the statistical analyses were those infants that had participated in the follow‐ups at both one and 13 years of age (see Figure 1).

^b^ Information about sex, siblings, and maternal smoking was collected at 1 year of age.

^c^ When the child was 1 year of age.

^d^ Information about heredity and cat/dog‐keeping was collected at 13 years of age.

^e^ Cat/dog‐keeping during the first year of life is regardless of cat/dog‐keeping after the first year of life.

^f^ Cat/dog‐keeping only after the first year of life, that is, no cat/dog‐keeping during the first year of life.

### 2.3 Skin‐prick test

Skin‐prick tests (SPTs) were performed at 1, 4, and 13 years of age. One nurse (always the same) performed all SPTs on the volar aspect of the lower arm at 1 (n = 1040) and 13 (n = 788) years of age. The test panel included cat, birch, timothy, egg, and milk at 1 year of age and was extended to also include dog, horse, soy, wheat, and fish at 13 years of age. Histamine dihydrochloride 10 mg/mL was used as a positive control. Children were instructed to avoid antihistamines for 72 hours before the test.

### 2.4 Definitions of exposure variables

*Dog‐ or cat‐keeping during the first year of life* was defined as dog or cat ownership during the child's first year, regardless of cat or dog ownership after the first year. This information was collected via questionnaires distributed at 13 years of age.

*Dog‐ or cat‐keeping after first year* was defined as dog or cat ownership any time during the period between one and 13 years of age, but not before 1 year of age. This information was collected via questionnaires distributed at 13 years of age.

### 2.5 Outcome definitions

*Sensitization* at 13 years of age was defined as a positive SPT for a specific allergen if the mean wheal diameter was at least 3 mm after 15 minutes.

*Hay fever* at 13 years of age was defined as a parental reported allergic reaction identified by itchy eyes and/or runny nose and sneezing upon contact with pollen allergens.

*Asthma* at 13 years of age was defined as parental reported respiratory symptoms during the last 12 months, and/or asthma medication, and/or asthma diagnosed by a doctor.

*Dog allergy* at 13 years of age was defined as parental reported symptoms of rhinoconjunctivitis or respiratory difficulties upon contact with dogs or dog allergens.

*Cat allergy* at 13 years of age was defined as parental reported symptoms of rhinoconjunctivitis or respiratory difficulties upon contact with cats or cat allergens.

### 2.6 Reverse causation

*Avoidance of pet keeping*, based on answers to the questionnaire administered at 13 years, was defined as an affirmative answer to the question: “Have you actively avoided pet keeping due to allergies or asthma in other family members?” All families that answered affirmatively (n = 177) were excluded from the logistic regression analyses in which cat‐ or dog‐keeping were used as exposure variables.

### 2.7 Statistical analyses

Our study is based on questionnaire responses from a prospective birth cohort study^10^, ^22^ in which data were coded and analyzed using SPSS statistics software (IBM SPSS statistics data editor, version 22). Odds ratios (OR) were calculated to study the association between exposure (cat‐ or dog‐keeping, paternal hay fever, paternal asthma, maternal hay fever, maternal asthma) and outcome (sensitization and occurrence of allergic diseases at 13 years of age). Statistical significance was set at the 5% probability level. The logistic regression analysis was carried out using both crude models without adjustments and models adjusted for the following potential covariates: paternal hay fever, paternal asthma, maternal hay fever, maternal asthma, maternal smoking, older siblings, sex, dog‐ and cat‐keeping. Sensitivity analyses based on parental allergy were performed for cat‐ and dog‐keeping during the first year of life to analyze a potential effect modification of maternal and/or paternal allergy on sensitization and allergy in the child. Maternal asthma and/or hay fever was reported for 221 of the 834 infants while paternal asthma or hay fever was reported for 209 of the infants. Any paternal or maternal asthma or hay fever was reported for 370 of the infants whereas 464 did not report parental allergy. Questions about heredity, smoking, and older siblings were asked at 1 year of age.

### 2.8 Ethical aspects

The Ethics Committee at the University of Umeå, Sweden, approved the study (Nr. 95‐149, 2009‐1116‐31 and 2012‐163‐32M). Written consent from the parents was required, and at 13 years of age, the child answered the questionnaire together with the parents and verbally approved all tests before proceeding with the SPTs.

## 3 RESULTS

### 3.1 Sensitization at 13 years of age

Sensitization to airborne allergens was common at 13 years of age (Table 2). The prevalence rate of at least one positive SPT at 13 years was 32%. The corresponding rates previously reported at 1 and 4 years were 7% and 13%, respectively.^10^

**Table 2:** Sensitization to 10 selected allergens at 13 years of age

Allergen	Positive SPT^a^	%
Number of tested subjects	788	100
Any allergens^b^	249	32
Timothy	161	19
Cat	140	17
Birch	133	16
Horse	97	12
Dog	81	10
Soy	9	1
Wheat	9	1
Fish	8	1
Egg	7	1
Milk	7	1

Abbreviation: SPT, skin‐prick test.

^a^ Number of children in the cohort with a positive SPT to the allergen.

^b^ Positive SPT to one or more of the 10 tested allergens.

### 3.2 Sensitization at 13 years of age in relation to dog‐ and cat‐keeping

Dog‐keeping during the first year of life reduced the risk of sensitization to dog allergen, adj. odds ratio (OR) 95% confidence interval (CI): 0.18 (0.08‐0.44), birch allergen (adj. OR [95% Cl]: 0.53 [0.29‐0.98]), and any allergen (adj. OR [95% Cl]: 0.58 [0.37‐0.92]). Dog‐keeping, not until after the first year of life, reduced the risk of dog and cat sensitization (Table 3).

**Table 3:** Sensitization to dog, cat, birch, and any allergen at 13 years of age in relation to the parental history of asthma and hay fever, and to pet ownership

	Pos SPT to dog at 13 y (n = 81)				Pos SPT to cat at 13 y (n = 140)			
	Crude OR (95% CI)	*P* crude	Adj OR^b^ (95% CI)	*P* adjusted	Crude OR (95% CI)	*P* crude	Adj OR^b^ (95% CI)	*P* adjusted
Paternal hay fever	2.51 (1.53‐4.10)	<.001	2.22 (1.30‐3.87)	.005	2.28 (1.51‐3.43)	<.001	2.42 (1.53‐3.85)	<.001
Paternal asthma	2.09 (1.15‐3.80)	.016	1.44 (0.73‐2.86)	.294	1.36 (0.80‐2.33)	.26	0.92 (0.50‐1.69)	.792
Maternal hay fever	2.08 (1.26‐3.42)	.004	1.78 (1.01‐3.14)	.047	2.20 (1.46‐3.29)	<.001	2.08 (1.31‐3.31)	.002
Maternal asthma	1.74 (0.98‐3.10)	.06	1.37 (0.70‐2.65)	.356	1.34 (0.82‐2.20)	.246	1.07 (0.61‐1.87)	.812
Dog‐keeping^c^								
Never	1.0		1.0		1.0		1.0	
First year of life^d^	0.20 (0.09‐0.46)	<.001	0.18 (0.08‐0.44)	<.001	0.56 (0.33‐0.96)	.034	0.61 (0.35‐1.06)	.081
After first year only^e^	0.47 (0.24‐0.94)	.032	0.38 (0.18‐0.82)	.014	0.55 (0.31‐0.98)	.044	0.47 (0.25‐0.88)	.018
Cat‐keeping^c^								
Never	1.0		1.0		1.0		1.0	
First year of life^d^	0.64 (0.32‐1.27)	.199	0.77 (0.37‐1.60)	.480	0.45 (0.24‐0.81)	.009	0.50 (0.27‐0.95)	.033
After first year only^e^	0.25 (0.10‐0.65)	.005	0.27 (0.10‐0.74)	.010	0.41 (0.22‐0.76)	.004	0.42 (0.22‐0.80)	.009

	Pos SPT to birch at 13 y (n = 133)				Pos SPT to any allergen^a^ at 13 y (n = 251)			
	Crude OR (95% CI)	*P* crude	Adj OR^b^ (95% CI)	*P* adjusted	Crude OR (95% CI)	*P* crude	Adj OR^b^ (95% CI)	*P* adjusted
Paternal hay fever	1.87 (1.23‐2.86)	.004	1.81 (1.12‐2.91)	.016	1.96 (1.38‐2.79)	<.001	1.87 (1.25‐2.79)	.002
Paternal asthma	1.28 (0.73‐2.22)	.392	0.84 (0.45‐1.60)	.603	1.40 (0.90‐2.19)	.140	1.07 (0.64‐1.78)	.804
Maternal hay fever	2.66 (1.77‐4.00)	<.001	2.12 (1.36‐3.43)	.001	2.50 (1.76‐3.55)	<.001	2.28 (1.53‐3.42)	<.001
Maternal asthma	1.87 (1.16‐3.01)	.010	1.36 (0.79‐2.35)	.262	1.65 (1.10‐2.48)	.017	1.18 (0.74‐1.89)	.489
Dog‐keeping^c^								
Never	1.0		1.0		1.0		1.0	
First year of life^d^	0.52 (0.29‐0.93)	.026	0.53 (0.29‐0.98)	.042	0.55 (0.36‐0.84)	.006	0.58 (0.37‐0.92)	.021
After first year only^e^	0.74 (0.42‐1.30)	.295	0.77 (0.43‐1.39)	.387	0.73 (0.47‐1.13)	.158	0.69 (0.43‐1.11)	.126
Cat‐keeping^c^								
Never	1.0		1.0		1.0		1.0	
First year of life	0.38 (0.20‐0.73)	.004	0.43 (0.22‐0.84)	.013	0.50 (0.32‐0.80)	.004	0.58 (0.35‐0.94)	.028
After first year only^e^	0.38 (0.19‐0.72)	.003	0.39 (0.20‐0.77)	.006	0.52 (0.33‐0.82)	.005	0.54 (0.33‐0.87)	.012

Abbreviations: CI, confidence interval; OR, odds ratio; Pos SPT, positive skin‐prick test.

^a^ At least one positive SPT when testing 10 different allergens at 13 years of age.

^b^ After multiple logistic regression adjusting for paternal hay fever, paternal asthma, maternal hay fever, maternal asthma, maternal smoking, older siblings, sex, and dog‐ or cat‐keeping.

^c^ After exclusion of all children whose families reported avoiding pet ownership during their first year of life because of asthma or allergies in other family members (n = 177).

^d^ Comparing cat/dog‐keeping during the first year of life (regardless of cat/dog‐keeping after the first year of life) with never cat/dog‐keeping from birth to 13 years of age.

^e^ Comparing cat/dog‐keeping after the first year of life with never cat/dog keeping from birth to 13 years of age.

Similarly, cat‐keeping during the first year of life reduced the risk of sensitization to cat allergen (adj. OR [95% CI]: 0.50 [0.27‐0.95]), birch allergen (adj. OR [95% CI]: 0.43 [0.22‐0.84]), and any allergen (adj. OR [95% CI]: 0.58 [0.35‐0.94]). Cat‐keeping, not until after the first year of life, reduced the risk for sensitization to the cat, dog, birch, and any allergen (Table 3).

### 3.3 Allergy at 13 years of age in relation to dog‐ and cat‐keeping

Dog‐keeping, during the first year of life, reduced the risk of both dog (adj. OR [95% CI]: 0.33 [0.14‐0.76]) and cat (adj. OR [95% CI]: 0.33 [0.17‐0.64]) allergy, whereas dog‐keeping, later in life, did not reduce the risk of allergy symptoms. Cat‐keeping, during the first year of life, reduced the risk of cat allergy (adj. OR [95% CI]: 0.43 [0.22‐0.85]) and hay fever (adj. OR [95% CI]: 0.40 [0.21‐0.78]). Cat‐keeping, after the first year of life, reduced the risk of dog allergy (adj. OR ([95% CI]: 0.35 [0.13‐0.93]), cat allergy (adj. OR [95% CI]: 0.38 [0.19‐0.77]), and hay fever (adj. OR [95% CI]: 0.30 [0.15‐0.62]; Table 4). Asthma at 13 years of age was not related to cat‐ or dog‐keeping at any time during childhood (Table 4). To take reverse causation into account all infants whose parents had answered that they actively avoided pet keeping due to allergies or asthma in other family members were excluded from the logistic regressions.

**Table 4:** Allergic symptoms at 13 years of age in relation to pet ownership, family history of asthma and hay fever (n = 834)

	Dog allergy 13 y (n = 76)				Cat allergy 13 y (n = 124)			
	Crude OR (95% CI)	*P* crude	Adj OR^a^ (95% CI)	*P* adjusted	Crude OR (95% CI)	*P* crude	Adj OR^a^ (95% CI)	*P* adjusted
Paternal hay fever	2.30 (1.34‐3.83)	.001	1.93 (1.08‐3.43)	.026	2.43 (1.560‐3.70)	<.001	2.14 (1.34‐3.34)	.002
Paternal asthma	1.85 (0.98‐3.45)	.054	1.24 (0.60‐2.57)	.555	1.84 (1.09‐3.09)	.022	1.29 (0.71‐2.34)	.398
Maternal hay fever	1.82 (1.08‐3.07)	.025	1.57 (0.86‐2.84)	.141	1.86 (1.21‐2.86)	.004	1.66 (1.02‐2.72)	.042
Maternal asthma	1.79 (0.99‐3.22)	.055	1.66 (0.86‐3.22)	.133	1.54 (0.93‐2.55)	.093	1.36 (0.77‐2.40)	.289
Dog‐keeping^b^								
Never	1.0		1.0		1.0		1.0	
First year of life^c^	0.33 (0.14‐0.74)	.007	0.33 (0.14‐0.76)	.009	0.32 (0.17‐0.61)	<.001	0.33 (0.17‐0.64)	.001
After first year^d^	0.50 (0.23‐1.11)	.087	0.45 (0.20‐1.04)	.062	0.66 (0.37‐1.17)	.153	0.59 (0.32‐1.08)	.087
Cat‐keeping^b^								
Never	1.0		1.0		1.0		1.0	
First year of life^c^	0.60 (0.28‐1.30)	.194	0.63 (0.28‐1.42)	.263	0.40 (0.21‐0.78)	.007	0.43 (0.22‐0.85)	.015
After first year^d^	0.32 (0.12‐0.86)	.023	0.35 (0.13‐0.93)	.035	0.40 (0.21‐0.78)	.007	0.38 (0.19‐0.77)	.007

	Hay fever 13 y (n = 155)				Asthma 13 y (n = 102)			
	Crude OR (95% CI)	*P* crude	Adj OR^a^ (95% CI)	*P* adjusted	Crude OR (95% CI)	*P* crude	Adj OR^a^ (95% CI)	*P* adjusted
Paternal hay fever	2.40 (1.60‐3.61)	<.001	2.31 (1.45‐3.66)	<.001	1.60 (1.04‐2.45)	.032	1.33 (0.81‐2.18)	.254
Paternal asthma	1.47 (0.87‐2.48)	.151	0.91 (0.49‐1.68)	.761	2.72 (1.68‐4.40)	<.001	2.49 (1.43‐4.35)	.001
Maternal hay fever	2.99 (2.01‐4.445)	<.001	2.87 (1.82‐4.52)	<.001	2.72 (1.82‐4.06)	<.001	1.96 (1.24‐3.12)	.004
Maternal asthma	1.9 (0.98‐2.57)	.060	1.05 (0.61‐1.84)	.852	3.86 (2.49‐5.98)	<.001	2.81 (1.69‐4.65)	<.001
Dog keeping^b^								
Never	1.0		1.0		1.0		1.0	
First year of life^c^	0.76 (0.45‐1.28)	.306	0.82 (0.47‐1.45)	.503	0.76 (0.45‐1.27)	.299	0.29 (0.04‐3.34)	.245
After first year^d^	0.64 (0.35‐1.16)	.141	0.6 7(0.36‐1.28)	.226	0.86 (0‐50‐1.49)	.593	0.89 (0.50‐1.61)	.712
Cat‐keeping^b^								
Never	1.0		1.0		1.0		1.0	
First year of life^c^	0.36 (0.19‐0.70)	.001	0.40 (0.21‐0.78)	.007	1.01 (0.60‐1.70)	.986	1.03 (0.58‐1.82)	0.028
After first year^d^	0.27 (0.13‐0.54)	<.001	0.30 (0.15‐0.62)	.001	0.78 (0.44‐1.36)	.375	0.68 (0.37‐1.26)	0.012

Abbreviations: CI, confidence interval; OR, odds ratio; SPT, skin‐prick test.

^a^ After multiple logistic regression adjusting for paternal hay fever, paternal asthma, maternal hay fever, maternal asthma, maternal smoking, older siblings, sex, and dog‐ or cat‐keeping.

^b^ After exclusion of all children whose families reported avoiding pet ownership during their first year of life because of asthma or allergies in other family members (n = 177).

^c^ Comparing cat/dog‐keeping during the first year of life (regardless of cat/dog‐keeping after the first year of life) with never cat/dog‐keeping from birth to 13 years of age.

^d^ Comparing cat/dog‐keeping after the first year of life with never cat/dog‐keeping from birth to 13 years of age.

The association between cat and dog‐keeping and allergy was similar in the crude unadjusted logistic regression models and in the logistic regression models adjusted for paternal hay fever, paternal asthma, maternal hay fever, maternal asthma, maternal smoking, older siblings, sex, and dog‐ and cat‐keeping (Table 4), which suggests that the association was present even when controlling for these factors.

To validate the effect of early cat‐ or dog‐keeping on sensitization and allergy further, sensitivity analyses were performed where the infants were stratified according to parental allergy. When comparing the number of infants with sensitization or allergy at 13 years of age according to early pet keeping the same associations were seen both for the 464 infants without, and for the 370 infants with, parental heredity to allergy. For example, in the group of infants with no parental allergy, among infants with no cat‐keeping 37 (16%) had hay fever at 13 years of age while among infants who had been exposed to a cat at home during the first year of life six (5%) had hay fever at 13 years of age. For the group of infants with paternal allergy, the numbers were 72 (31%) vs 8 (13%), respectively.

### 3.4 Sensitization and allergy at 13 years of age in relation to maternal and paternal heredity

Parental hay fever doubled the risk of sensitization to dog, cat, birch, and to any allergen at 13 years of age (Table 3). This is in contrast to parental asthma which did not significantly alter the risk of sensitization at 13 years of age (Table 3). Finally, parental hay fever increased the risk for the child to develop dog allergy, cat allergy, and hay fever (Table 4) opposed to parental asthma with no significant altered risk of allergic reactions to dog, cat, or hay fever but more than doubled the risk for the child to develop asthma (Table 4).

## 4 DISCUSSION

Our results suggest that having a dog or a cat at home during the first year of life, as well as later during childhood, can reduce the risk of sensitization to common airborne allergens at age 13. Also, having a dog at home during the first year of life may reduce the risk of dog and cat allergy, whereas cat‐keeping, both during and after the first year of life, may reduce the risk of cat allergy and hay fever at 13 years of age.

The association between dog‐ or cat‐keeping during the first year of life and a lower risk of sensitization to these specific allergens at age 13 might partly be explained by the exposure to a high dose of dog and cat allergens early in life. This exposure may influence the immune system during an important time period, called “window of opportunity”, in which tolerance can be induced.^24^ However, the time and mechanism by which tolerance was induced in our subjects cannot be further speculated on since we have no additional immunological analyses.

Having a dog or cat at home during the first year of life also reduced the risk of sensitization to birch allergens at 13 years of age which is in line with our previous report from the same study population where we described an association between dog‐keeping during the first year of life and a reduced risk of sensitization to pollen at 4 years of age.^10^ Both these observations from our prospective cohort study are consistent with results of other longitudinal studies.^8^, ^13^, ^14^ Having dogs or cats at home may, in addition to allergen load, affect other exposures for substances in the indoor environment such as different bacteria and rate of microbial turnover. It has been shown that indoor environments with dogs have higher concentrations of endotoxins.^25^ Exposure to endotoxins and other microbial compounds has been suggested to reduce the risk of atopic symptoms and of sensitization among children,^26^ especially in families with cats and dogs.^27^ This is in accordance with another recent observation on early microbial exposure: a reduced risk of asthma and eczema was observed in children whose parents cleaned the child's pacifier in their own mouth, when dropped on the floor, before giving it back to the child.^1^ The authors explained that this reduced risk may be due to the immune stimulation by microbes transferred to the infant via the parent's saliva. A similar microbial impact could be a possible explanation in an environment with indoor pets and reduced risk of sensitization due to positive stimulation of the immune system. This hypothesis is in line with a recent study on early pet keeping and later allergy development, showing that the protective effect from cats and dogs is dose‐dependent, suggesting that cats and dogs induce an allergy‐protective “mini‐farm” environment.^28^ How bacterial and other microbes may modulate immunological activities and reduce the risk of sensitization and allergic symptoms need to be further elucidated.

Dog‐ and cat‐keeping during infancy affected the risk of self‐reported dog or cat allergy at age 13. In addition, cat‐keeping during the first year of infancy, but not dog‐keeping during the first year, reduced the risk of hay fever, a highly hereditary condition induced by pollen from both grass and trees. Parents with hay fever may hesitate to accept a cat or a dog at home and to be able to avoid reverse causation we removed all families who reported avoiding pet ownership due to allergy symptoms among other family members from our analyses. To further validate the effect of parental heredity on the association between early cat‐ or dog‐keeping on sensitization and allergy, we divided the infants into those with no parental heredity to hay fever or asthma and those with either maternal and/or paternal hay fever or asthma and compared the associations between pet keeping and sensitization and allergy. The associations were similar in the two groups suggesting that the effect of early life cat or dog‐keeping on sensitization and allergy is not modified by parental heredity to allergy.

Other studies have reported conflicting results regarding the effects of dog‐ and cat‐keeping on sensitization or allergy development during childhood. In one study of 399 children, dog‐ and cat‐keeping during the third trimester of pregnancy or the first year of life did not show any association with sensitization or rhinitis.^29^ In another study with pooled data from 11 prospective studies, the presence of dogs or cats at home did not affect the risk of asthma or allergic rhinitis in children.^30^ One recent study reported a higher incidence of dog and cat allergy during the first 4 years of life in children with early exposure to the respective animal during infancy.^31^ This is in contrast to our findings of lower rates of sensitization and allergy symptoms with either a cat or dog in the home during the child's first year and also with a cat in the home later in childhood but not during infancy.

It is well known that a parental history of allergy is an important risk factor for asthma, allergic symptoms, and sensitization.^32^, ^33^ In this study, we found that parental asthma increased the risk of asthma in the children, while parental hay fever increased their risk of hay fever, cat allergy, and for paternal hay fever even dog allergy.

This study has several strengths, including the prospective birth cohort design with longitudinal follow‐up at specific time‐points, the total area coverage with inclusion of all children born at Östersund Hospital during a 1‐year period, the use of validated questionnaires, and the fact that all SPT were carried out by the same nurse. A limitation of our study may be that allergy diagnosis was made using parent‐reported questionnaire‐based symptoms. However, the surveys were based on questions from the ISAAC study,^23^ that is, validated and used in numerous studies over many years.

In conclusion, having a dog or a cat at home during the first year of life reduced the risk of sensitization and allergic symptoms to dogs and cats, respectively, whereas having a cat also reduced the risk of sensitization and allergic symptoms to birch allergen and hay fever. Cat‐keeping later during childhood, although not dog‐keeping, reduced the risk of dog allergy, cat allergy, and hay fever. Although this is in line with several earlier studies, more research is needed to understand the mechanisms behind the allergy‐protective effects associated with the keeping of cats and dogs, early in life.

## DATA ACCESSIBILITY

The data that support the findings of this study are available from the corresponding author upon reasonable request.
